# Supplementary material for: Leveraging large multi-center cohorts of Alzheimer disease endophenotypes to understand the role of Klotho heterozygosity on disease risk
Source: PLoS One. 2022 May 26;17(5):e0267298. doi: 10.1371/journal.pone.0267298 (PMC9135221; doi:10.1371/journal.pone.0267298)
Supplement: S1 File — (DOCX) [file pone.0267298.s001.docx]

Figure S1. KL-VS heterozygosity status is associated with CSF Aβ42 levels in cognitively normal participants.


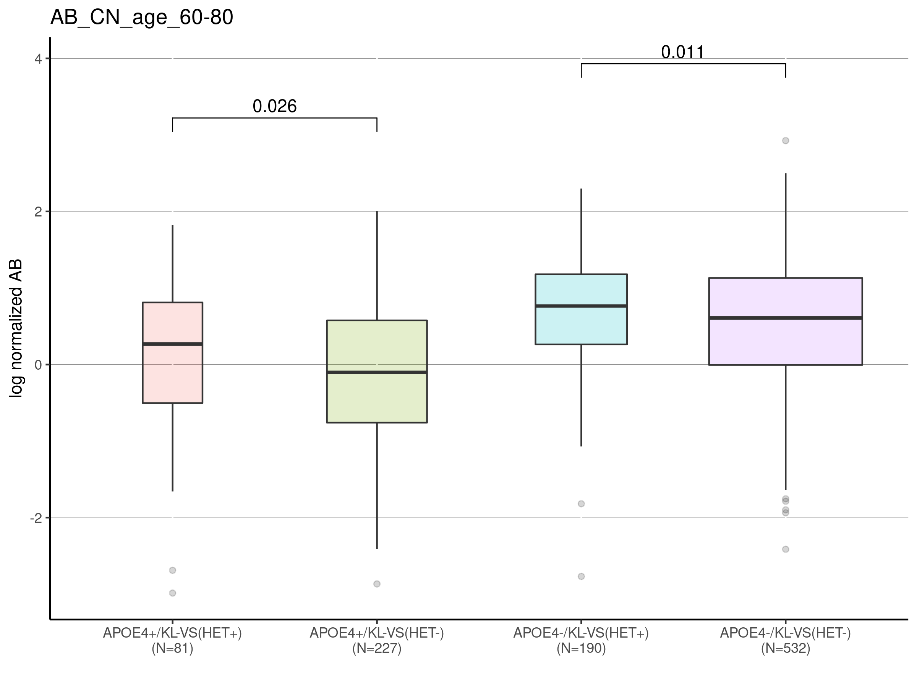


A significant association was detected between Klotho-VS^HET+^ and dichotomized CSF amyloid beta42 (Aβ) levels. The distribution of quantitative log-normalized CSF Aβ42 levels are shown in this box plot, stratified by *APOE* ε4 and KL-VS^HET+^ status, where width of the boxplot represents the sample size of each group. Box plot error bars show the 95th-percentile range. Gray circles indicate values outside of the 95th percentile range. There is a significant difference between the means of compared groups (*APOE*4+/ KL-VS^HET+^ vs. *APOE*4+/ KL-VS^HET-^ and *APOE*4-/ KL-VS^HET+^ vs. *APOE*4-/ KL-VS^HET-^) with mean comparison p-values labelled on the top. Abbreviations: *APOE*4+, *Apolipoprotein E*4 positive; KL-VS^HET+^, Klotho-VS heterozygous; KL-VS^HET-^, Klotho-VS homozygous; N, number of samples.

Figure S2: First two principal components of the genetic population structure across 17 cohorts together with the reference HapMap data (CEU, JPT, and YRI).


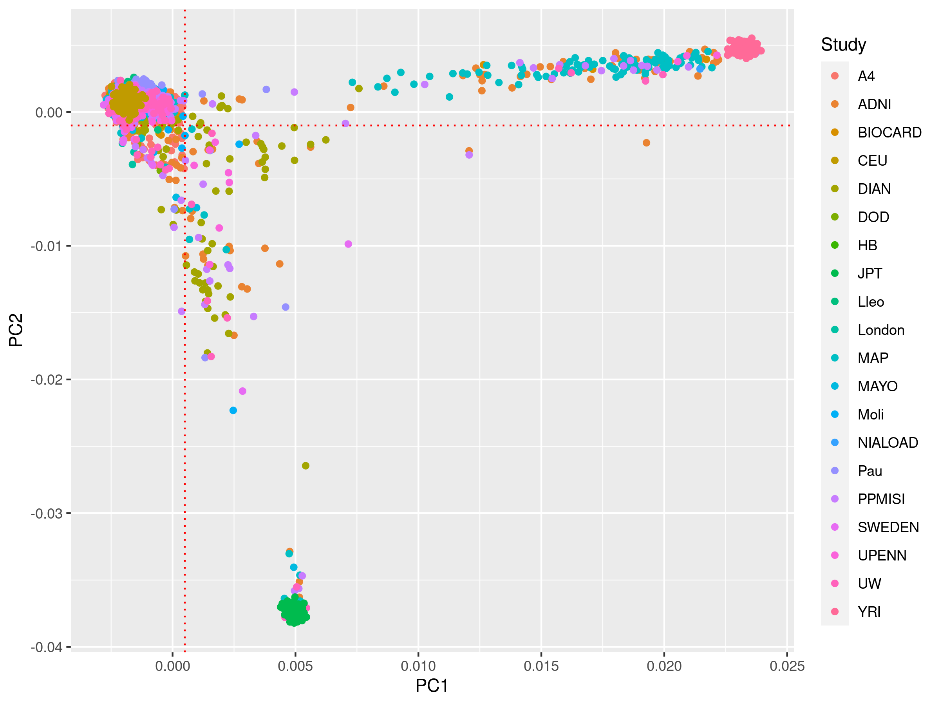


First two principal components of the genetic population structure across 17 cohorts (A4, ADNI, BIOCARD, DIAN, ADNIDOD, HB, Lleo, London, MAP, MAYO, Moli, NIALOAD, Pau, PPMISI, SWEDEN, UPENN, and UW) analyzed in this study, together with reference HapMap data (CEU, JPT, and YRI). The red dotted lines represent the thresholds (PC1 < 0.0005 and PC2 > -0.0010) for defining 9,526 European ancestry participants. Abbreviations: PC, principal component.

Figure S3: First three principal components of the genetic population structure in European ancestry participants across all 17 cohorts.


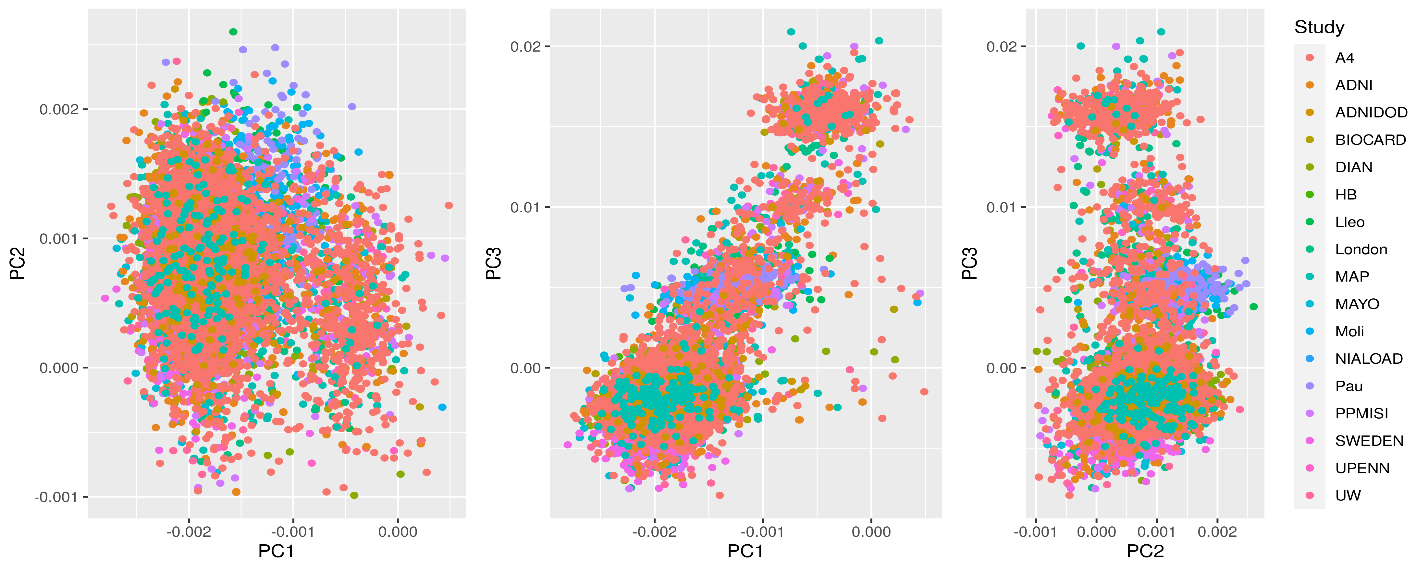


First three principal components (PCs) of the genetic population structure across 17 cohorts (A4, ADNI, BIOCARD, DIAN, DOD, HB, Lleo, London, MAP, MAYO, Moli, NIALOAD, Pau, PPMI, SWEDEN, UPENN, and UW) analyzed in this study. Abbreviations: PC, principal component.
