# Supplementary material for: Leveraging large multi-center cohorts of Alzheimer disease endophenotypes to understand the role of Klotho heterozygosity on disease risk
Source: PLoS One. 2022 May 26;17(5):e0267298. doi: 10.1371/journal.pone.0267298 (PMC9135221; doi:10.1371/journal.pone.0267298)
Supplement: S2 File — (DOCX) [file pone.0267298.s002.docx]

| **Table S1. Demographic characteristics for each analyzed cohort.** | | | | | | | | | | | | | | | |
| --- | --- | --- | --- | --- | --- | --- | --- | --- | --- | --- | --- | --- | --- | --- | --- |
| **Cohort name** | **Participants** | | | **Diagnosis % (AD/CN)** | | | **Age, mean (sd)** | | | **Sex % (F/M)** | | | ***APOE*4+ (%)** | | |
|  | **All** | **60-80** | **CN** | **All** | **60-80** | **CN** | **All** | **60-80** | **CN** | **All** | **60-80** | **CN** | **All** | **60-80** | **CN** |
| **MAP** | 1323 | 954 | 565 | 27/61 | 29/59 | 0/100 | 70 (10) | 71 (5) | 70 (5) | 54/46 | 53/47 | 55/45 | 41 | 44 | 35 |
| **NIALOAD** | 24 | 15 | 14 | 17/79 | 7/93 | 0/100 | 74 (9) | 72 (5) | 71 (5) | 62/38 | 60/40 | 57/43 | 21 | 27 | 29 |
| **ADNI** | 1734 | 1368 | 401 | 63/27 | 61/29 | 0/100 | 74 (7) | 72 (5) | 71 (5) | 45/55 | 46/54 | 56/44 | 44 | 46 | 29 |
| **BIOCARD** | 215 | 97 | 53 | 0/69 | 0/55 | 0/100 | 58 (10) | 65 (5) | 64 (4) | 60/40 | 53/47 | 60/40 | 33 | 30 | 26 |
| **DIAN** | 411 | 17 | - | 8/6 | 0/0 | - | 39 (11) | 63 (3) | - | 56/44 | 53/47 | - | 30 | 41 | - |
| **HB** | 102 | 71 | - | 100/0 | 100/0 | - | 68 (9) | 70 (6) | - | 45/55 | 2/58 | - | 53 | 56 | - |
| **Lleo** | 137 | 81 | 39 | 36/64 | 52/48 | 0/100 | 64 (10) | 70 (6) | 68 (4) | 65/35 | 64/36 | 64/36 | 36 | 41 | 31 |
| **London** | 249 | 192 | 18 | 27/8 | 26/9 | 0/100 | 69 (9) | 70 (6) | 65 (4) | 46/54 | 46/54 | 28/72 | 47 | 44 | 33 |
| **Moli** | 232 | 155 | 31 | 56/22 | 56/20 | 0/100 | 65 (8) | 68 (6) | 68 (6) | 63/37 | 59/41 | 39/61 | 37 | 37 | 16 |
| **Pau** | 143 | 106 | - | 12/0 | 12/0 | - | 67 (9) | 70 (5) | - | 50/50 | 47/53 | - | 36 | 35 | - |
| **MAYO** | 400 | 219 | 181 | 6/76 | 4/83 | 0/100 | 79 (6) | 76 (3) | 76 (3) | 38/62 | 42/58 | 43/57 | 28 | 31 | 27 |
| **SWEDEN** | 284 | 194 | - | 0/0 | 0/0 | - | 75 (8) | 74 (5) | - | 63/37 | 58/42 | - | 76 | 77 | - |
| **UPENN** | 113 | 80 | - | 0/0 | 0/0 | - | 72 (9) | 72 (5) | - | 58/42 | 51/49 | - | 58 | 68 | - |
| **UW** | 355 | 197 | - | 0/0 | 0/0 | - | 63 (16) | 70 (6) | - | 50/50 | 49/51 | - | 43 | 48 | - |
| **PPMISI** | 546 | 316 | 87 | 0/29 | 0/28 | 0/100 | 62 (10) | 68 (5) | 68 (5) | 33/67 | 29/71 | 28/72 | 27 | 25 | 26 |
| **A4** | 3106 | 2935 | 2934 | 0/100 | 0/100 | 0/100 | 71 (5) | 71 (4) | 71 (4) | 60/40 | 61/39 | 61/39 | 36 | 37 | 37 |
| **ADNIDOD** | 152 | 147 | 98 | 32/68 | 33/67 | 0/100 | 69 (5) | 69 (4) | 69 (4) | 1/99 | 1/99 | 1/99 | 27 | 27 | 30 |
| Demographics of participants at the time of amyloid PET imaging and CSF sampling across each cohort. This table summarizes the demographic characteristics of participants for each analyzed cohort. Participant stratification was performed at three levels, 1) All, All of the participants; 2) 60-80, Participants who are 60 to 80 years old; 3) CN, Participants who are cognitively normal and aged 60 to 80 years. For each cohort, we report number of participants in each stratum, their diagnosis (number of AD and cognitively normal participants), mean age of the participants and standard deviation in the age across cohort, percentage of females and males, and percentage of APOE4+ participants. Abbreviations: %, percentage; AD, Alzheimer's Disease; CN, cognitively normal; sd, standard deviation; F, Females; M, Males; *APOE*4+, *Apolipoprotein E*4 positive; All, All participants (controls and AD); 60-80: Participats aged 60-80 (controls and AD); CN: 60-80, cognitively normal participants aged 60-80; MAP, Memory and Aging Project (MAP); NIALOAD, National Institute on Aging Late Onset Alzheimer's Disease; ADNI, Alzheimer's Disease Neuroimaging Initiative; BIOCARD; DIAN, the Dominantly Inherited Alzheimer Network; HB; Lleo; London; MOLI; Pau; MAYO, Mayo Clinic; SWEDEN; UPENN; UW; PPMI, Progression Markers Initiative; A4, Anti-Amyloid Treatment in Asymptomatic Alzheimer's Disease; ADNIDOD, ADNI Department of Defense. | | | | | | | | | | | | | | | |

| **Table S2. Empirical dichotomization cutoffs derived from z-scores and their corresponding raw values.** | | | |
| --- | --- | --- | --- |
| **Cohort** | **Modality** | **Z-score threshold** | **Raw value threshold** |
| MAP | Aβ (PET) | 0.55 | 33.01 |
| ADNI |  | 0.43 | 48.93 |
| MAP | Aβ42 (CSF) | -0.33 | 527.30 |
| ADNI |  | 0.29 | 185 |
| A Gaussian mixture model (GMM) was used to statistically infer the dichotomization cut-off based on the normalized z-scores for each endophenotype. The raw endophenotypic value corresponding each dichotomization threshold is also provided in the table. Abbreviations: Aβ, β-amyloid; PET, positron emission tomography; MAP, Memory and Aging Project; ADNI, Alzheimer's Disease Neuroimaging Initiative; CSF; Cerebrospinal fluid. | | | |

| **Table S3. Genetic association of KL-VS^HET+^ with dichotomized AD endophenotypes, stratified by *APOE* ε4 status.** | | | | | |
| --- | --- | --- | --- | --- | --- |
| Modality | Group | CN/AD participants  (KL-VS^HET+^ %) | Odds ratio | Estimate | P value |
| Amyloid PET | **All participants** |  | | | |
|  | APOE4+ | 1432/526 (27.2/24) | 0.89 | -0.12 | 0.26 |
|  | APOE4- | 2685/564 (26.4/25.5) | 1.02 | 0.02 | 0.85 |
|  | **Age 60-80** |  | | | |
|  | APOE4+ | 1328/421 (27.5/25.9) | 0.87 | -0.14 | 0.23 |
|  | APOE4- | 2397/371 (26.5/26.1) | 1.02 | 0.02 | 0.84 |
|  | **CN: Age 60-80** |  | | | |
|  | APOE4+ | 1328/NA (27.5/NA) | 0.94 | -0.07 | 0.61 |
|  | APOE4- | 2397/NA (26.5/NA) | 0.99 | -0.01 | 0.90 |
| AB42 | **All participants** |  | | | |
|  | APOE4+ | 461/1076 (26.7/24.9) | 0.55 | 0.20 | 0.07 |
|  | APOE4- | 1123/1348 (26.3/25.7) | 0.52 | 0.07 | 0.43 |
|  | **Age 60-80** |  | | | |
|  | APOE4+ | 308/814 (26.3/25.9) | 0.56 | 0.24 | 0.08 |
|  | APOE4- | 722/921 (26.3/24.9) | 0.54 | 0.16 | 0.15 |
|  | **CN: Age 60-80** |  | | | |
|  | APOE4+ | 308/NA (26.3/NA) | 0.67 | 0.72 | **0.007** |
|  | APOE4- | 722/NA (26.3/NA) | 0.61 | 0.46 | **0.03** |
| Tau | **All participants** |  | | | |
|  | APOE4+ | 465/1095 (27.3/24.7) | 1.07 | 0.07 | 0.51 |
|  | APOE4- | 1124/1348 (26.3/25.3) | 0.86 | -0.11 | 0.27 |
|  | **Age 60-80** |  | | | |
|  | APOE4+ | 308/828 (26.9/25.6) | 1.02 | 0.02 | 0.89 |
|  | APOE4- | 722/920 (26.5/24.5) | 1.01 | 0.01 | 0.95 |
|  | **CN: Age 60-80** |  | | | |
|  | APOE4+ | 308/NA (26.9/NA) | 0.39 | -0.94 | **0.007** |
|  | APOE4- | 722/NA (26.5/NA) | 0.85 | -0.16 | 0.49 |
| pTau | **All participants** |  | | | |
|  | APOE4+ | 465/1024 (27.3/24) | 0.96 | -0.04 | 0.73 |
|  | APOE4- | 1117/1273 (26.2/25.3) | 0.98 | -0.02 | 0.88 |
|  | **Age 60-80** |  | | | |
|  | APOE4+ | 308/779 (26.9/24.8) | 0.91 | -0.09 | 0.48 |
|  | APOE4- | 722/864 (26.3/24.5) | 1.04 | 0.04 | 0.76 |
|  | **CN: Age 60-80** |  | | | |
|  | APOE4+ | 308/NA (26.9/NA) | 0.50 | -0.68 | **0.04** |
|  | APOE4- | 722/NA (26.3/NA) | 0.89 | -0.11 | 0.61 |
| sTREM2 | **All participants** |  | | | |
|  | APOE4+ | 274/569 (28.8/24.1) | 0.96 | -0.04 | 0.78 |
|  | APOE4- | 605/505 (25/27.5) | 0.89 | -0.11 | 0.42 |
|  | **Age 60-80** |  | | | |
|  | APOE4+ | 199/469 (31.2/25.4) | 0.89 | -0.11 | 0.53 |
|  | APOE4- | 440/378 (25.2/26.2) | 0.99 | -0.01 | 0.96 |
|  | **CN: Age 60-80** |  | | | |
|  | APOE4+ | 199/NA (31.2/NA) | 1.08 | 0.08 | 0.80 |
|  | APOE4- | 440/NA (25.2/NA) | 1.20 | 0.18 | 0.43 |
| Association between KL-VS^HET+^ and different dichotomized AD endophenotypes were assessed using logistic regression model. Association were assessed across three strata: 1) all participants (controls and AD), 2) participants aged 60–80 (controls and AD), and 3) cognitively normal participants aged 60–80 years. We used dichotomized endophenotype as the response variable, whereas, age, sex, and first three genetic PCs were used as covariates in an *APOE* ε4-stratified analysis. Significant associations are represented by bold P-values. Abbreviations: KL-VS^HET+^, Klotho-VS heterozygous; CN, cognitively normal; AD, Alzheimer’s disease; Std. Error, Standard error; %, percentage; Aβ, β-amyloid; pTau, phosphorylated tau; soluble triggering receptor expressed on myeloid cells 2, sTREM2. | | | | | |

| **Table S4. Genetic association of KL-VS^HET+^ with AD endophenotypes in cognitively normal participants aged 60–80 years (*APOE4*-interaction model).** | | | | |
| --- | --- | --- | --- | --- |
| **Modality** | **CN participants**  **(KL-VS^HET+^ %)** | **Odds ratio** | **Estimate** | **P value** |
| **Amyloid PET** | 1328 (27.5) | 0.94 | -0.06 | 0.71 |
|  |  |  |  |  |
| **Aβ42** | 308 (26.3) | 1.30 | 0.27 | 0.43 |
|  |  |  |  |  |
| **Tau** | 308 (26.9) | 0.48 | -0.74 | 0.07 |
|  |  |  |  |  |
| **pTau** | 308 (26.9) | 0.60 | -0.50 | 0.20 |
|  |  |  |  |  |
| **sTREM2** | 199 (31.2) | 0.88 | -0.13 | 0.73 |
| Association between KL-VS^HET+^ and different dichotomized AD endophenotypes were assessed using logistic regression model. We used dichotomized endophenotype as the response variable, whereas, age, sex, and first three genetic PCs were used as covariates in an *APOE*4-interaction model. Significant associations are represented by bold P-values. Abbreviations: KL-VS^HET+^, Klotho-VS heterozygous; CN, cognitively normal; AD, Alzheimer’s disease; Std. Error, Standard error; %, percentage; Aβ, β-amyloid; pTau, phosphorylated tau181; soluble triggering receptor expressed on myeloid cells 2, sTREM2. | | | | |

| **Table S5. Genetic association of** **homozygous KL-VS haplotype (KL-VS^HET-^) with dichotomized AD endophenotypes, stratified by *APOE* ε4 status.** | | | | | |
| --- | --- | --- | --- | --- | --- |
| Modality | Group | CN/AD participants  (KL-VS^HET-^ %) | Odds ratio | Estimate | P value |
| Amyloid PET | **All participants** |  | | | |
|  | APOE4+ | 1042/400 (96.9/94.2) | 0.90 | -0.11 | 0.73 |
|  | APOE4- | 1976/420 (96.4/96.4) | 0.76 | -0.28 | 0.22 |
|  | **Age 60-80** |  | | | |
|  | APOE4+ | 963/312 (96.8/94.6) | 1.07 | 0.06 | 0.84 |
|  | APOE4- | 1761/274 (96.4/96.4) | 0.73 | -0.32 | 0.21 |
|  | **CN: Age 60-80** |  | | | |
|  | APOE4+ | 963/NA (96.8/NA) | 1.09 | 0.08 | 0.83 |
|  | APOE4- | 1761/NA (96.4/NA) | 0.69 | -0.37 | 0.18 |
| AB42 | **All participants** |  | | | |
|  | APOE4+ | 338/808 (96.4/97.2) | 1.19 | 0.18 | 0.60 |
|  | APOE4- | 828/1002 (95.9/97.4) | 1.21 | 0.19 | 0.42 |
|  | **Age 60-80** |  | | | |
|  | APOE4+ | 227/603 (96.5/96.8) | 0.97 | -0.03 | 0.93 |
|  | APOE4- | 532/692 (95.7/97.1) | 1.27 | 0.24 | 0.42 |
|  | **CN: Age 60-80** |  | | | |
|  | APOE4+ | 227/NA (96.5/NA) | 0.95 | -0.05 | 0.95 |
|  | APOE4- | 532/NA (95.7/NA) | 1.46 | 0.38 | 0.41 |
| Tau | **All participants** |  | | | |
|  | APOE4+ | 338/825 (96.4/97.0) | 0.71 | -0.34 | 0.23 |
|  | APOE4- | 828/1007 (95.9/97.2) | 1.19 | 0.18 | 0.53 |
|  | **Age 60-80** |  | | | |
|  | APOE4+ | 225/616 (96.4/96.6) | 0.69 | -0.37 | 0.26 |
|  | APOE4- | 531/695 (95.7/96.8) | 1.01 | 0.01 | 0.98 |
|  | **CN: Age 60-80** |  | | | |
|  | APOE4+ | 225/NA (96.4/NA) | 0.80 | -0.22 | 0.77 |
|  | APOE4- | 531/NA (95.7/NA) | 0.88 | -0.13 | 0.82 |
| pTau | **All participants** |  | | | |
|  | APOE4+ | 338/778 (96.4/97.4) | 1.01 | 0.01 | 0.98 |
|  | APOE4- | 824/951 (95.9/97.4) | 0.93 | -0.07 | 0.79 |
|  | **Age 60-80** |  | | | |
|  | APOE4+ | 225/586 (96.4/96.9) | 0.83 | -0.18 | 0.60 |
|  | APOE4- | 532/652 (95.7/97.1) | 0.93 | -0.07 | 0.84 |
|  | **CN: Age 60-80** |  | | | |
|  | APOE4+ | 225/NA (96.4/NA) | 1.48 | 0.39 | 0.64 |
|  | APOE4- | 532/NA (95.7/NA) | 0.76 | -0.27 | 0.61 |
| sTREM2 | **All participants** |  | | | |
|  | APOE4+ | 195/432 (97.4/97.0) | 0.80 | -0.21 | 0.66 |
|  | APOE4- | 454/366 (95.8/97.3) | 0.84 | -0.17 | 0.66 |
|  | **Age 60-80** |  | | | |
|  | APOE4+ | 137/350 (97.1/96.9) | 1.11 | 0.10 | 0.85 |
|  | APOE4- | 329/279 (96.0/97.1) | 0.79 | -0.24 | 0.61 |
|  | **CN: Age 60-80** |  | | | |
|  | APOE4+ | 137/NA (97.1/NA) | 6.12 | 1.81 | 0.13 |
|  | APOE4- | 329/NA (96.0/NA) | 0.54 | -0.62 | 0.33 |
| Association between homozygous KL-VS haplotype (KL-VS^HET+^) and different dichotomized AD endophenotypes were assessed using logistic regression model. Association were assessed across three strata: 1) all participants (controls and AD), 2) participants aged 60–80 (controls and AD), and 3) cognitively normal participants aged 60–80 years. We used dichotomized endophenotype as the response variable, whereas, age, sex, and first three genetic PCs were used as covariates in an *APOE* ε4-stratified analysis. KL-VS^HET-^ (%) represents the percentage of samples containing 2/0 copies of KL-VS. Abbreviations: KL-VS^HET-^, Klotho-VS homozygous; CN, cognitively normal; AD, Alzheimer’s disease; Std. Error, Standard error; %, percentage; Aβ, β-amyloid; pTau, phosphorylated tau; soluble triggering receptor expressed on myeloid cells 2, sTREM2. | | | | | |

| **Table** **S6. Genetic association of KL-VS^HET+^ with AD endophenotypes in cognitively normal age- and sex-matched participants, stratified by *APOE* ε4 status.** | | | | | |
| --- | --- | --- | --- | --- | --- |
| **Modality** | **Group** | **CN participants**  **(KL-VS^HET+^ %)** | **Odds ratio** | **Estimate** | **P value** |
| **Amyloid PET** | APOE4+ | 1324 (27.6) | 0.93 | -0.07 | 0.57 |
|  | APOE4- | 1324 (25.5) | 0.95 | -0.05 | 0.72 |
|  |  |  |  |  |  |
| **Aβ42** | APOE4+ | 307 (26.1) | 0.68 | 0.75 | **0.005** |
|  | APOE4- | 307 (26.1) | 0.67 | 0.70 | **0.03** |
|  |  |  |  |  |  |
| **Tau** | APOE4+ | 307 (26.7) | 0.39 | -0.93 | **0.008** |
|  | APOE4- | 307 (26.1) | 0.49 | -0.71 | 0.11 |
|  |  |  |  |  |  |
| **pTau** | APOE4+ | 307 (26.7) | 0.51 | -0.67 | **0.04** |
|  | APOE4- | 307 (26.1) | 0.75 | -0.28 | 0.45 |
|  |  |  |  |  |  |
| **sTREM2** | APOE4+ | 192 (29.7) | 1.15 | 0.14 | 0.66 |
|  | APOE4- | 192 (23.4) | 1.08 | 0.08 | 0.82 |
| Association between KL-VS^HET+^ and different dichotomized AD endophenotypes were assessed using logistic regression model. We used dichotomized endophenotype as the response variable, whereas, age, sex, and first three genetic PCs were used as covariates in an *APOE* ε4-stratified analysis. For each modality, we considered equal number of age- and sex-matched participants from both *APOE* ε4 strata. Significant associations are represented by bold P-values. Abbreviations: KL-VS^HET+^, Klotho-VS heterozygous; CN, cognitively normal; AD, Alzheimer’s disease; Std. Error, Standard error; %, percentage; Aβ, β-amyloid; pTau, phosphorylated tau; soluble triggering receptor expressed on myeloid cells 2, sTREM2. | | | | | |
