# Supplementary material for: Leveraging large multi-center cohorts of Alzheimer disease endophenotypes to understand the role of Klotho heterozygosity on disease risk
Source: PLoS One. 2022 May 26;17(5):e0267298. doi: 10.1371/journal.pone.0267298 (PMC9135221; doi:10.1371/journal.pone.0267298)
Supplement: Attachment — Submitted filename: Response_to_Reviewers.docx [file pone.0267298.s003.docx]

*Dear Reviewers,*

*We are immensely thankful for your insightful comments and suggestions. We believe your suggestions will improve the quality of the manuscript, therefore, we have addressed all the comments/suggestions as per the best of our understanding and available data. For further details, please see the answers to the specific questions/comments below:*

**Reviewer 1:**

**Minor revision:**
1) There is no uniformity in the way papers are cited: for example, references #8, 12, 17, 35, 36, and 52. Concerning reference 56, publication date is wrong: 2014 > 1997. Please carefully check the section of References again.

*Answer: All of the references have been carefully corrected and standardized as per the format of the journal.*

2) Please correct the word "KL-VSHET+" to superscript (KL-VS^HET+^) in Table 2.

*Answer: Corresponding text has been changed in the table title (see track changes).*

3) I wonder if a description of Figure S1 is not able to find.

*Answer: This figure was not referenced in the manuscript text.* *We have created a reference to this image in line 287 on page 13 (see track changes).*

4) It is helpful for readers to present representative brain imaging data (i.e., amyloid PET images) of KL-VS^HET+^ to easily understand the protective impact of the variants in the main body of the manuscript or a supplementary figure.

*Answer: Unfortunately, we do not have access to the imaging data. We only have access to the raw mean SUVR scores for each sample; therefore, we are unable to provide this information.*

**Reviewer 2:**

1. The authors mentioned in the introduction that the hetero group with one copy of KL-VS is more protective of the brain than the homo group with two copies of KL-VS (KL-VSHET-). Intuitively, I thought that having more KL-VS haplotypes would be important for brain protection, but this does not seem to be the case. Although the authors examined only the group with 1 copy of KL-VS in this study, I am also interested in the association between APOEε4 and AD pathologies in the group with 0 copies of KL-VS and the group with 2 copies of KL-VS.

*Answer: We have assessed the associations between APOE4 and AD pathologies in the group with 2 and 0 copies of KL-VS haplotype (KL-VS^HET-^). Results showed no significant association across any AD endophenotypes (Table S5), confirming the existing hypothesis that suggests the neuroprotective effect of KL heterozygosity (KL-VS^HET+^) but not the homozygosity (KL-VS^HET-^). An additional supplementary table (Table S5) and corresponding text has been added in the manuscript (lines 305 – 313; see track changes).*

2. In Table 2, the authors found a significant decrease in CSF Tau and pTau in APOEε4 carriers compared to APOEε4 non-carriers. To show more clear statistical results, I recommend that the authors perform an association analysis including an interaction effect for the presence or absence of APOE4 and the status of each biomarker (positive or negative) and include the statistics in the paper.

*Answer: We have used an updated model where APOE4 interaction effect was included with the status (positive or negative) for each biomarker. The general linear model used for this analysis is:*

*glm(Bin.score ~ Sex + Age + PC1 + PC2 + PC3 + rs9536314*APOE4+)*

*Where Bin.Score represent dichotomized biomarker status and APOE4+ represents the binarized APOE4 status (0 = APOE-, 1= APOE4+). By using the described interaction effect model we did not observed any significant association between* *KL-VS^HET+^ and available endophenotypes. Text explaining these results has been added in the manuscript (line 309-3**13; see track changes) and a new supplementary table (Table S4) has been added.*

3. Figure S1 is not included in the text.
*Answer: We have created a reference to this image in line 287 (see track changes).*

4. The PCA plot in Figure S3 should have a smaller plot size and be color-coded by the study as in Figure S2.

*Answer: We have created a new image in which points are color-coded by the study (Figure S3).*
